# Supplementary material for: Protective Effect of Lactiplantibacillus plantarum 1201 Combined with Galactooligosaccharide on Carbon Tetrachloride-Induced Acute Liver Injury in Mice
Source: Nutrients. 2021 Dec 12;13(12):4441. doi: 10.3390/nu13124441 (PMC8706614; doi:10.3390/nu13124441)
Supplement: Supplementary file 1 [file nutrients-13-04441-s001.zip › nutrients-1485103-supplementary.pdf]

Supporting Information

Table S1 Primer sequences for quantitative Real-Time polymerase chain reaction.

Gene		Sequence
β-actin	Forward	GCTCCTCCTGAGCGCAAGTA
	Reverse	CAGCTCAGTAACAGTCCGCC
IκB-α	Forward	GAAGAGAAGCCGCTGACCAT
	Reverse	CAGAAGTGCCTCAGCAATTCC
NF-κB	Forward	ACGATCTGTTTCCCCTCATC
	Reverse	TGCTTCTCTCCCCAGGAATA
IL-22	Forward	CCGAGGAGTCAGTGCTAAGG
	Reverse	TCTGGATGTTCTGGTCGTCA
IL-1β	Forward	ACAGGCTCCGAGATGAACAAC
	Reverse	GTGGGTGTGCCGTCTTTCAT
IL-6	Forward	CTGCAAGAGACTTCCATCCAG
	Reverse	AGTGGTATAGACAGGTCTGTTGG
IFN-γ	Forward	TGATTGCGGGGTTGTATCTG
	Reverse	CTGTCTGGCCTGCTGTTAAA
IL-17A	Forward	CTCCAGAAGGCCCTCAGACTA
	Reverse	AGCTTTCCCTCCGCATTGACA
TNF-α	Forward	CCCTCACACTCAGATCATCTTCT
	Reverse	GCTACGACGTGGGCTACAG
TGF-β1	Forward	GTCACTGGAGTTGTACGGCA
	Reverse	TCATGTCATGGATGGTGCCC
IL-10	Forward	GCTCTTACTGACTGGCATGAG
	Reverse	CGCAGCTCTAGGAGCATGTG
Ocln	Forward	GGACTGTCAACTCTTTCCGC
	Reverse	CATTTATGATGAACAGCCCC
Cldn3	Forward	CTGTCTGTCCTCTTCCAGCC
	Reverse	CCACTACCAGCAGTCGATGA
CCL4	Forward	GAAACAGCAGGAAGTGGGAG
	Reverse	CATGAAGCTCTGCGTGTCTG
CCL5	Forward	CCACTTCTTCTCTGGGTTGG
	Reverse	GTGCCCACGTCAAGGAGTAT
FN-1	Forward	ATGAGAAGCCTGGATCCCCT
	Reverse	GGAAGGGTAACCAGTTGGGG
ZO-1	Forward	GATCCCTGTAAGTCACCCAGA
	Reverse	CTCCCTGCTTGCACTCCTATC
